# Miscellany

**Published:** 1904-05

**Authors:** 


### Miscellany.

---

Local Analgesia by Adrenalin-Eucaine.—Barker (*Lancet,* July 25, 1903) *naïvely* remarks that this method of abolishing pain has, with little doubt, a future before it. He states that he believes, now that the principles which underlie it are well understood, it will be widely employed. Full credit is given to Corning for his admirable work in this direction, and attention is called to the fact that because of the rapid absorption of the injected material no operation lasting more than twenty minutes can be performed without a constant reinfiltration of the tissues and nerves.

Beta-eucaine he notes is less toxic than cocaine; both these drugs have the following drawbacks: (1) The difficulty of injecting directly through the nerve-trunk and through the skin; (2) the need of employing a large quantity of the solution of 1 to 500 to thoroughly anæsthetize a region supplied by several nerve-trunks.

It is well known that anything which retards or diminishes the circulation of the blood in a part infiltrated with an analgesic agent enhances the potency of the latter. This fact suggested the possibility of increasing the local anæsthetic effect of eucaine by the injection of adrenalin, which would necessarily diminish the stream of blood in the part. It was noted that after a lapse of about twenty minutes the part thus injected with the combined drugs was quite blanched and wholly insensitive to pain, and that this loss of feeling lasted on an average about two hours. Barker has been so well satisfied with the result of his first test that he has employed the combination for several months, and with results far superior to those produced by the eucaine alone. His method of preparing the solution is as follows: To one hundred cubic centimetres of boiled sterile water 0.2 gramme of beta-eucaine and 0.8 gramme of sodium chloride are added; thereafter eighteen minims of adrenalin chloride (Parke, Davis & Co.) are dropped from the stoppered bottle in which this drug comes into the eucaine solution. If the bottle be corked at once, it will not spoil. This makes a solution of 1 to 500 eucaine and 1 to 100,000 of adrenalin chloride.

There were no instances of secondary hemorrhage, though a number of operations, such as those for the radical cure of hernia, were successfully performed.—*Therapeutic Gazette.*

